# The Laboratory Diagnosis of Malaria: A Focus on the Diagnostic Assays in Non-Endemic Areas

**DOI:** 10.3390/ijms25020695

**Published:** 2024-01-05

**Authors:** Adriana Calderaro, Giovanna Piccolo, Carlo Chezzi

**Affiliations:** Department of Medicine and Surgery, University of Parma, Viale A. Gramsci 14, 43126 Parma, Italy; giovanna.piccolo@unipr.it (G.P.); carlo.chezzi@unipr.it (C.C.)

**Keywords:** *Plasmodium* sp., imported malaria, diagnosis, molecular methods, polymerase chain reaction, RTD, microscopy, flow cytometry

## Abstract

Even if malaria is rare in Europe, it is a medical emergency and programs for its control should ensure both an early diagnosis and a prompt treatment within 24–48 h from the onset of the symptoms. The increasing number of imported malaria cases as well as the risk of the reintroduction of autochthonous cases encouraged laboratories in non-endemic countries to adopt diagnostic methods/algorithms. Microscopy remains the gold standard, but with limitations. Rapid diagnostic tests have greatly expanded the ability to diagnose malaria for rapid results due to simplicity and low cost, but they lack sensitivity and specificity. PCR-based assays provide more relevant information but need well-trained technicians. As reported in the World Health Organization Global Technical Strategy for Malaria 2016–2030, the development of point-of-care testing is important for the improvement of diagnosis with beneficial consequences for prompt/accurate treatment and for preventing the spread of the disease. Despite their limitations, diagnostic methods contribute to the decline of malaria mortality. Recently, evidence suggested that artificial intelligence could be utilized for assisting pathologists in malaria diagnosis.

## 1. Introduction

Malaria, from the Italian words “mal aria” meaning “unhealthy air”, is still a health problem in the world. Five species of parasites can infect humans, namely *Plasmodium falciparum* (*Pf*), *Plasmodium vivax* (*Pv*), *Plasmodium malariae* (*Pm*), *Plasmodium ovale curtisi* (*Poc*), *Plasmodium ovale wallikeri* (*Pow*), and *Plasmodium knowlesi* (*Pk*), even if *P. cynomolgi*, *P. brasilianum* and *P. simium* cases in Southeast Asia and in South America have been described [1,2,3,4]. Malaria is a potentially fatal mosquito-borne parasitic disease and its clinical presentation, known for many centuries, is the cause of suffering and a high number of deaths globally. *P. falciparum* is responsible for more than 90% of the world’s malaria mortality remaining an important threat to public health [5] followed by *P. vivax* accounting for 75% of infections and representing the most common species in the World Health Organization (WHO) regions of Americas [6].

Malaria is endemic in more than 90 countries with an estimated 247 million cases and 619,000 deaths globally in 2021 [7]. The number of imported malaria cases and indigenous cases following the imported ones is also increasing in non-endemic areas [5]. Imported malaria cases are mostly diagnosed in travelers and migrants from endemic areas and their clinical management requires attention because of non-specific symptoms at the onset, difficulties related to the laboratory diagnosis due to low parasitemia, and treatment possibilities due to potential drug resistance [8]. The goal of the WHO Global Technical strategy for Malaria, by 2030, is the reduction of the incidence and mortality rates by 90%. This should help to stop malaria transmission in at least 35 countries and is considered a way to prevent malaria re-establishment in all malaria-free-countries [9].

The development of new strategies for malaria prevention includes insecticides and vaccines, and single dose drugs.

The WHO reported among malaria vectors a spread of global resistance to pyrethroids that are the most used insecticide-treated mosquito nets and a less prevalent resistance to carbamates and organophosphates [9]. This suggests a rising incidence of malaria in the areas where such a phenomenon was registered as new insecticides are not yet commercially available. Thus, efforts in vector controls are required to make malaria control possible in endemic areas.

Together with vector control strategies, vaccines were developed. In some regions of sub-Saharan Africa, a vaccine acting against *P. falciparum* is currently in a phase 3 trial and it was recommended by the WHO as a complementary malaria control tool that could be added to (and not replace) the preventive, diagnostic and treatment measures recommended by the WHO. This is the only vaccine that has demonstrated to be able to significantly reduce malaria, and life-threatening severe malaria, in young African children, but it is not protective against *P. vivax* malaria, which is more prevalent outside of Africa [9].

The first line treatment of uncomplicated malaria is the oral artemisinin-based combination therapy (ACT) or parenteral artesunate in severe malaria [9]. The adoption, since the 2000s, of ACT together with an improved parasitological diagnosis contributed to the decline of malaria related mortality globally, and particularly in non-endemic areas. It is clearly demonstrated that accurate diagnosis and prompt effective treatment of malaria also prevents severe sequelae and death and reduces the risk of onward transmission of the parasite in malaria endemic areas.

Malaria symptoms are nonspecific consisting of fever, fatigue, myalgia, abdominal pain, nausea, vomiting, diarrhea, chills, headache and altered mentation [10] that might cause an incorrect clinical diagnosis. For this reason, appropriate diagnostic methods are required to differentiate malaria from other febrile diseases. In a febrile patient returning from a malaria-endemic country, malaria should be always suspected [11] considering that in subjects with no or low immunity uncomplicated *P. falciparum* malaria can rapidly evolve to complicated clinical stages of the disease, and severe *P. falciparum* malaria could be fatal without a prompt and appropriate treatment. Programs for malaria control should guarantee fast access to prompt diagnosis and effective anti-malaria treatment as soon as possible and no later than 24–48 h from the onset of malaria symptoms.

A prompt and accurate malaria diagnosis can prevent the worsening of the disease and the spread of the malaria parasites [7] and can reduce the severity of the disease, especially for kids under 5 years of age, which was the cause of about 80% of deaths in 2021 due to severe malaria in Africa [7]. The identification of the involved species of *Plasmodium* and the number of parasites in the blood (parasitemia) is essential to set up an adequate treatment of malaria; in fact, parasitemia is one of the criteria to define severe malaria. Patient management should change in case of parasitemia >2% and in case of detection of mature asexual forms (>20% of parasites); in fact, the parasitemia contributes to the definition of *P. falciparum* severe malaria [12].

Once the diagnosis of malaria has been made, the identification of the causative *Plasmodium* species is necessary to administer an appropriate therapy that should be initiated as soon as possible. Generally, admission to the hospital is recommended for malaria cases [7].

Due to the great and increasing number of imported malaria cases and the consequent risk of reappearance of indigenous cases, many laboratories in non-endemic areas had to carefully evaluate adopted diagnostic algorithms and methods. In a non-endemic setting, a skilled microscopist is not always present, especially when the diagnosis of malaria is required in emergencies during laboratory closing hours or in areas far from a laboratory [13,14].

The present review reports a systematic analysis of the different methodologies currently available for the diagnosis of malaria in laboratories located in non-endemic areas.

## 2. Diagnostic Assays

Malaria diagnoses should be confirmed by laboratory assays and t patients should be promptly directed to a facility with diagnostic capabilities [15].

### 2.1. Microscopy

Microscopy is still considered the gold standard for malaria diagnosis, despite advances in diagnostic technologies in the past 20 years. In all clinical settings according to the WHO, a malaria diagnosis must be performed by microscopic examination of Giemsa-stained thin and thick blood smears for *Plasmodium* sp. identification and parasitemia count or rapid diagnostic tests (RDTs) [16]. Two slides of each type must be performed to increase the diagnostic yield and all cases identified by laboratory-confirmed diagnosis should be reported to the State Health Department. Microscopy should be performed instantly, and results should be available as soon as possible and no later than 2 h from sampling, in ≤24 h of the patient’s presentation [17]. In case of an initial negative result at microscopy, blood smears should be repeated at each febrile attack every 12–24 h for a total of three sets before the diagnosis of malaria can be excluded [18]. Thick blood films are mostly used to detect the presence of malaria parasites and to assess the parasitemia, while thin blood films are useful to identify the *Plasmodium* sp. and the circulating stages of the parasite’s life cycle stages within the blood of the patient [10]. Especially in areas where infection is not endemic and where malaria cases occur with low parasitemia, the parasite count can also be assessed by analyzing a well-stained thin blood film using microscopy as a percentage of infected red blood cells (RBC) [19]. The advantages of light microscopy include (a) low direct costs (2,5–5 Euros, excluding the cost of the microscope) in a high-volume sample; (b) good sensitivity and results in 2 h; (c) identification of *Plasmodium* sp. and stage differentiation; (d) parasitemia count; (e) drug-induced morphological changes observation; (f) the absence of parasites to assess the clearance of the plasmodia; (g) screening for other related blood abnormalities and other blood parasites (i.e., *Babesia*, *Trypanosoma*, *Filaria*) at once [16]. Parasitemia is essential for the classification of malaria severity and prognosis: a parasite density of more than 5% is a criterion to identify severe malaria cases and parasite density counting should be continued until parasites are cleared as a follow-up to evaluate the response to the anti-malaria treatment [18].

Unfortunately, microscopy has several limitations: (a) it cannot differentiate the morphology of all the stages between *P. knowlesi* and *P. malariae*; (b) it cannot differentiate the morphology of the early ring trophozoites between *P. knowlesi* and *P. falciparum* [20]; (c) many parasites can be missed during the staining procedure bringing to both a reduced sensitivity of the method and an incorrect count of the parasite density; (d) it has a limited analytic sensitivity causing a microscopic threshold of 50 parasites/µL and does not diagnose mixed infections; (e) it requires the availability of experienced laboratory personnel, particularly microscopists. The ring stages of *Plasmodium* parasites might be confused with the same stages of another protozoan parasite of the red blood cells, *Babesia*, or conversely, by an untrained examiner, often causing misdiagnosis [21].

**Figure 1:**
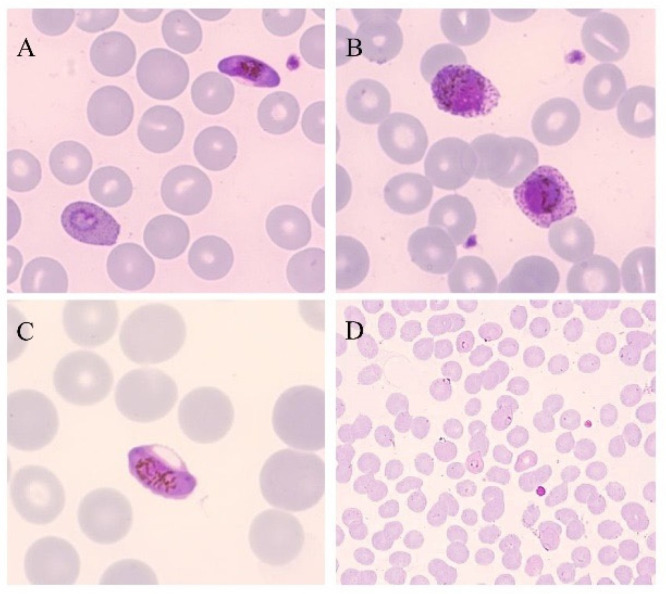
Thin blood smears of blood samples from malaria cases prepared and stained with Giemsa. (**A**) *P. falciparum* gametocyte and *P. ovale* trophozoite (100×). (**B**) *P. ovale* gametocytes (100×). (**C**) *P. falciparum* gametocyte (100×). (**D**) *P. falciparum* trophozoites (40×). (modified from [14]).

Microscopy is the only diagnostic tool able to demonstrate the presence of an active infection based on stage identification, in fact, considering the life cycle of human *Plasmodia* parasites, it can indicate “live” parasites. For this, microscopy is still considered the gold standard method for the laboratory diagnosis malaria (Figure 1), although currently, instruments that are able to automatically analyze the blood of patients have been developed and tested [22]. Moreover, automatic slide reading instruments or vision-based devices have also been developed and tested, even if microscopy by trained personnel is still the preferred approach [22]. In a study conducted in 2012, an automated malaria slide scanning system, the World Health Technology (WHT) autoanalyzer, was one of first systems tested performing at a level comparable to many human slide readers [23]. Cella Vision DM96 is another digital system that is applied on blood films and using its advanced red blood cell application (ARBCA), it is able to recognize and classify the cell morphology of both leukocytes and erythrocytes including parasitized erythrocytes [24].

**Figure 2:**
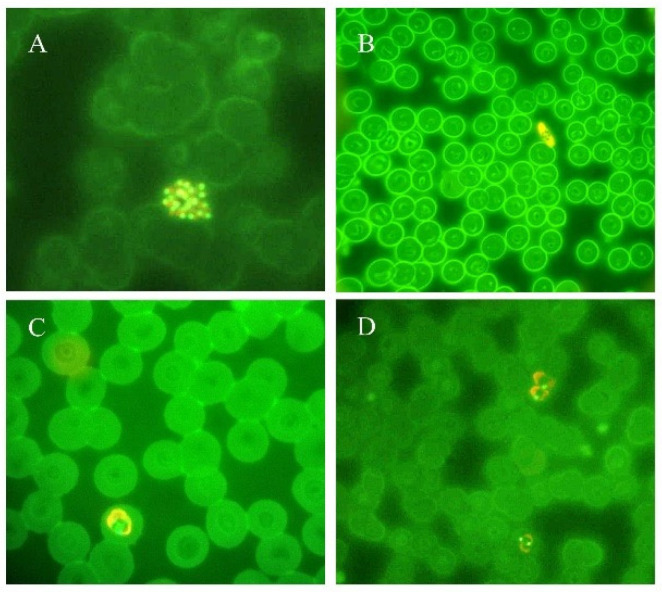
Thin blood smears of blood samples from malaria cases prepared and stained with acridine orange. (**A**) *P. vivax* schizont (100×). (**B**) *P. falciparum* gametocyte (40×). (**C**) *P. ovale* trophozoite (100×). (**D**) *P. vivax* trophozoites (100×). (modified from [14]).

The microscopic examination of *Plasmodium* parasites using parasite fluorescent labeling is another diagnostic procedure applied to the diagnosis of malaria: acridine orange staining is incubated with the patient’s blood and the DNA/RNA of the different stages of *Plasmodium* sp. is marked in green and orange, respectively [25]. Fluorescent parasites are successively detected by a conventional fluorescence microscope (Figure 2). The advantages of this approach are field applications because of a reduced energy requirement, stronger brightness, and contained costs. This approach allows better results compared to Giemsa staining under a revised acridine orange staining protocol [26]. Even if this is a feasible method which leads to fast diagnostic results in less than 1 h, trained personnel are needed to correctly label the patient’s blood samples and to correctly perform the analysis by fluorescent microscope [27]. However, the result of acridine orange staining must be confirmed with Giemsa staining.

### 2.2. Rapid Diagnostic Tests

In establishing prompt malaria diagnosis, multiple rapid diagnostic tests (RDTs) have been developed as a complementary test, providing a result within 15 min, and requiring minimal training. According to WHO recommendations [28], in areas where microscopy or other approaches are not available, antigen based RDTs can be a valid alternative to obtain a fast and easy diagnosis of malaria and for these reasons they are often adopted in health care systems to screen patients with clinically suspected malaria, followed by microscopy.

Malaria RDTs are lateral flow immunochromatographic tests on nitrocellulose strips which detect either species-specific or genus-specific *Plasmodium* sp. antigens or a combination of both in a finger-prick blood sample. RDTs allow the diagnosis of *P. falciparum* or *P. falciparum* versus non-*P*. *falciparum* infections. However, the non-*P. falciparum* malaria parasites generally cannot be revealed. Different formats of RDTs are commercially available, e.g., dipsticks, cassettes, and cards. Cassettes and cards are the easiest to use when health facilities are not available. RDTs are easy to perform and simple to interpret, not requiring equipment and they were originally suggested as kit for first diagnostic aid in travelers to endemic areas [29,30,31].

The results of the WHO malaria RDT product testing program, in 2012 produced the WHO recommendations that specifically indicated the selection of RDTs according to the following criteria that are reported unmodified from in the original WHO text [28]: “in all transmission settings for the detection of *P. falciparum*, the recommended panel detection score for *P. falciparum* samples should be at least 75% at 200 parasites/µL; in all transmission settings for the detection of *P. vivax*, the panel detection score against *P. vivax* samples should be at least 75% at 200 parasites/µL and false positive rates should be less than 10% and invalid rates less than 5% on the whole” [28].

Antigens commonly detected in commercially available RDTs are: 1. *P. falciparum*-specific antigen Histidine-Rich Protein 2 (HRP2); 2. a pan-*plasmodium* Lactate Dehydrogenase (LDH) (pan-pLDH); 3. *P. falciparum*-specific LDH (*Pf*LDH); 4. *P. vivax*-specific LDH (*Pv*LDH); 5. aldolase, which is also a pan-*plasmodium* antigen [32]. In Table 1 some commercially available RDTs and the parasite species detected are reported.

**Table 1:** Commercially available RDTs and parasite species detected.

	Species Tested					
	*P. falciparum*	*P. vivax*	*P. ovale*	*P. malariae*	* Pan	References
MalaQuick (R-Biopharm, Pfungstadt, Germany)	X				X	[33]
BinaxNOW^TM^ MALARIA (Abbott^TM^, Italy)	X				X	[34]
Clearview^®^ malaria (Orgenics Ltd., Alere Diagnostics, Yavne, Israel)	X	X	X	X		[35]
Carestart^TM^ Malaria (AccessBio Inc., Somerset, NJ, USA)	X	X	X	X		[36]
SD Bioline Malaria Ag 05FK40 (Standard Diagnostics Inc., Hagal-dong, Republic of Korea)	X					[37]
SD Bioline Malaria Ag *Pf* FK50 (Standard Diagnostics Inc., Republic of Korea)	X					[38]
SD FK70 Malaria Antigen *Pv* test (Standard Diagnostics Inc., Republic of Korea)		X				[39]
SD FK80 *Pf*/*Pv* Malaria Antigen Rapid Test (Standard Diagnostics Inc., Republic of Korea)	X	X				[39]
SD Malaria Antigen *Pf* 05FK90-02-0 (Standard Diagnostics, Inc., Republic of Korea)	X					[40]
VIKIA Malaria (Biomerieux, Marcy-l’Étoile, France)	X				X	[41]
Core Malaria (Core Diagnostics, Bromborough, UK)	X	X			X	[40]
PALUTOP^®^+4 OPTIMA (BioSynex, Illkirch-Graffenstaden, France)	X	X			X	[35]
OptiMal-IT^®^ (DiaMed, Cressier, Switzerland)	X				X	[35]
Immunoquick+4 (BioSynex, France)	X	X	X	X		[42]

All studies were performed in non-endemic areas, and the tests were carried out on symptomatic patients returning from endemic areas. * Pan: Pv/Pm/Po.

HRP2 is a protein produced only by *P. falciparum*, mainly by asexual stages and gametocytes, and RDTs based on it allow the benefit of *Pf* specificity together with a high sensitivity. HRP2 is used in over 80% of all RDTs and for this reason it is commonly chosen in Africa, where 99.7% of *P. falciparum* malaria cases occur [28]. RDT-*Pf*HRP2 has a 95% sensitivity and a 95.2% specificity [32]; however, at low parasitemia level (<1000 parasites/µL), the result can be interpreted as false negative due to a weak signal on the reaction’s line [32]. False negatives can also occur with gene deletions of HRP2, and this represents a limitation in the use of HRP2 based RDTs as tests of cure due to persistent antigenemia [32]. Moreover, false-positive results due to a cross-reaction with rheumatoid factor were rarely reported in the past [14].

All species of malaria parasites can be detected by the pLDH assays developed with the PpanLDH or more specifically with *Pf*LDH or *Pv*LDH and in such cases most of the limitations related to gene deletions or prozone seen with HRP2 can be avoided [32]. Furthermore, pLDH is much more effective as a test of cure having a specificity of 87% after treatment improving to 92–100% between days 7–42 [43]. PpanLDH has also proved be able to identify *P. knowlesi* with a 97% sensitivity at parasitemia >1000 parasites/µL, but only 25% when parasitemia is <1000 parasites/µL [32]. Overall, *Pf*-pLDH showed a 93.2% sensitivity and 98.5% specificity. It was demonstrated in *P. vivax*, that pan-pLDH versus *Pv*-pLDH has no difference and high sensitivity (>99%).

Aldolase-detecting RDTs still give a low sensitivity (80–81.4%) and they are based on this enzyme found in the glycolytic pathway of all species of malaria parasites [44].

The main limitations of RDTs together with the risk of false positive and false negative results, include their inability to quantify the parasitemia, to distinguish among the parasitic stages and the potential missing of double infections [14].

RDTs also have potential disadvantages: for the *Pf*HRP2-based RDTs, of interest is the inability to allow to distinguish new infections from those effectively treated and those recently acquired, related to the *Pf*HRP2 persistence in the blood for 1–5 weeks after an effective therapy; poor sensitivity in *P. malariae* and *P. ovale* detection and the heterogeneous quality of commercially available products producing the existence of batch-to-batch variation [31]. Another weakness of the RDTs is the positive results in non-malaria febrile patients [45]. A newly developed highly sensitive RDT (HS-RDT) represents a promising tool to better detect *Plasmodium* species in the blood of infected subjects [46].

**Figure 3:**
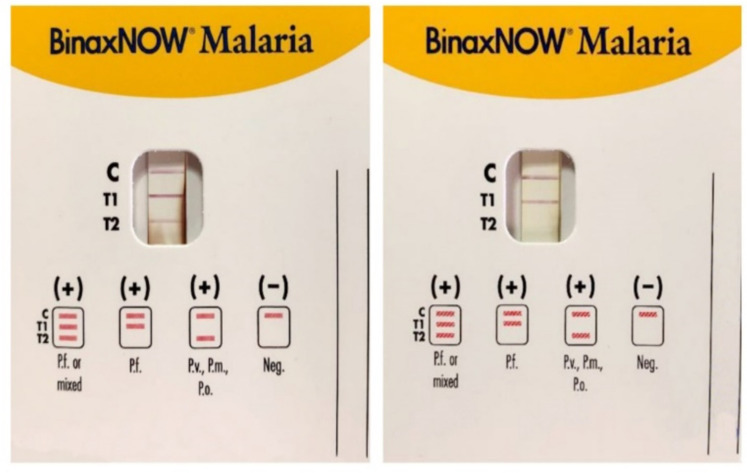
Immunocromatographic assay for the detection of *Plasmodium* sp. antigens in blood samples: *P. falciparum* (*Pf*), *P. malariae* (*Pm*), *P. vivax* (*Pv*), and *P. ovale* (*Po*). C is the control line for human blood, T1 line specific to *P. falciparum* (*Pf*) Histidine-Rich Protein2 (HRP2), and T2 line is for the parasite lactate aldolase. A *Pf* or mixed infection on the left and a *Pf* infection on the right (modified form [14]).

The United States Food and Drug Administration has approved only one RDT (BinaxNow^TM^), a card combining HRP2/Aldolase with a 95.3% sensitivity, and 94.2% specificity for *P. falciparum* [28] and 68.9–74.6%, and 99.8% for *P. vivax*, respectively. BinaxNOW^TM^ RDT has a *P. falciparum* line linked to HRP2 (T1), and a pan-malaria line (*Pv*, *Po*, or *Pm*) linked to aldolase T2 (Figure 3). When the result is the appearance of both T1 and T2 lines, it cannot be used alone to distinguish whether this is the case of a multi-species infection involving *P. falciparum* mixed with a non-*falciparum* species or the case of a high *Pf* parasitemia because the aldolase is a preserved enzyme in all species of malaria parasites, including *P. falciparum*.

The originally proposed use of RDTs was as a tool for self-diagnosis in high-risk groups, especially travelers in malaria endemic areas after appropriate training allowing timely an adequate management and avoiding over-diagnosis of malaria on-site and inappropriate antimalaria treatment. This use of RDTs is still controversial, although recent studies have produced encouraging results.

### 2.3. Flow Cytometry: Hemozoin-Based Diagnosis

Hemozoin (Hz), a pigment derived from the digestion of the host’s hemoglobin by the intra-erythrocytic stages of malaria parasites, is used as marker for the diagnosis of malaria by flow cytometry [47]. The method provides an 82–97% specificity and a 49–98% sensitivity, thus it could be used for the diagnosis of malaria, including cases clinically unsuspected [21]. Hz-containing leukocytes indicate the presence of *Plasmodium* sp. having a prognostic relevance in malaria; however, the detection of only a single pigmented leukocyte is highly indicative of malaria. Although different study sites produced highly variable results, most studies established a highly significant, positive correlation with the severity of the disease [21]. Some disadvantages are the need for trained technicians, its labor intensiveness, false positives with bacterial/viral infections, and expensive diagnostic equipment. Thus, this method should be considered as a potential tool for malaria screening [48]. In Table 2 methods based on the different characteristics of hemozoin are summarized.

**Table 2:** Methods for the laboratory diagnosis of malaria based on hemozoin characteristics.

Technology	Limit of Detection	References
Electromagnetic		
Magnetic Resonance Relaxometry (MRR)	0.002% of *Pf* culture	[49]
Microfluidic separation followed saponin lysis and MRR	0.0005% of *Pf* culture	[50]
Saponin lysis and MRR	0.0001% of *Pf* culture	[51]
Magneto-optic		
Magneto-optical technology	50–100 *Pf* culture/µL	[52]
Rotating-crystal magneto-optical technique	40–10 *Pf* culture /µL	[53]
Magneto-chromatographic online system	55 parasites (*Pf*)/µL	[54]
Gazzelle	50 parasites (*Pf*)/µL	[55]
Portable optical diagnostic system	25 parasites (*Pf*)/µL	[56]
Surface-enhanced Raman spectroscopy	30 parasites (*Pf*)/µL	[57]
Optical features		
Polarized light microscopy	30 parasites (*Pf*)/µL	[58]
Third-Harmonic Generation Imaging	Non-defined	[59]
Optical Absorbance Diagnostic Method	100%sensitivity–96.3%specificity until 1 µg hemozoin	[60]
Optical Reflectance Diagnostic Method	12 parasites (*Pf*)/µL	[61]
Polymerization-based Assay	10 parasites (*Pf*)/µL	[62]
Photoacoustic properties		
In vivo photoacoustic flow cytometry	less than 5 *P.yoelii*-infected mice/µL	[63]
In vivo photoacoustic flow cytometry	5 *P.yoelii*-infected mice/µL	[64]
Hemozoin-generated vapor nanobubbles	5 parasites (*Pf*)/µL	[65]
Photoacoustic excited surface acoustic wave	1000 parasites (*Pf*)/µL	[66]

All studies were performed in non-endemic areas and the tests carried out on symptomatic patients returning from endemic areas.

### 2.4. Serodiagnosis

Serological tests to search for the presence of anti-*Plasmodium* sp. antibodies in serum samples might be applied for the detection of *Plasmodium*-specific antibodies in epidemiological surveys and in the screening procedures of potential blood solid organ/cells donors who are natives/coming from endemic areas, but they are not recommended as a diagnostic approach for active malaria [67]. The immunofluorescence antibody test (IFAT) has been developed as a reliable serological assay for the detection of anti-*Plasmodium* sp. antibodies [68]. The concentration of immunoglobulin G/M in serum samples can be determined using fluorescence microscopy on *Plasmodium* derived antigens prepared on a slide. Another method to detect *Plasmodium*-specific antibodies in the patient’s serum/plasma is the enzyme-linked immunosorbent assay (ELISA) using different antigens derived from the different *Plasmodium* species in a 96-well plate and an appropriate plate reader [69]. These two methods are expensive and very time consuming and require trained personnel to both conduct the assay and analyze the results albeit they are relatively simple and moderately sensitive (84.2%) [68].

Because of the time needed to the development of detectable antibodies from the immune system of infected subjects and the persistence of antibodies in cured malaria cases, serologic testing is not applicable for the diagnosis of acute malaria. However, the serodiagnosis may be useful for several applications: (1) screening blood donors coming or natives from malaria endemic counties; (2) preventing induced malaria in case of the donor’s parasitemia below the detectable level of blood film microscopic examination; (3) testing a patient, usually from an endemic area, with tropical splenomegaly syndrome, a clinical condition observed in patients with a history of repeated or chronic malaria infections; (4) testing a patient with a recently treated malaria with uncertain diagnosis [70].

### 2.5. Molecular Methods

Due to the reasons described above, microscopy still remains the reference method for the laboratory diagnosis of malaria, while RDTs represent an important diagnostic aid over more traditional methods and molecular methods are currently used as confirmatory assays. In fact, molecular methods are crucial when the morphological characteristics of the parasites overlap each other, or parasite morphology is altered by drug treatment, in case of mixed infections by different *Plasmodium* species, incorrect storage of the samples, or when sub-microscopic parasitemia occurs [14,21,71].

Overall, nucleic acid amplification tests (NAATs) are at least 10-fold more sensitive compared to microscopy having a detection limit of about 0.2–6 parasites/µL of blood, based on the assay and the *Plasmodium* sp. involved [14]. The overall category of NAATs used to detect different *Plasmodium* sp. in the blood includes PCR (nested-PCR, multiplex-PCR, real-time PCR), loop-mediated isothermal amplification (LAMP), molecular-based point of care test (POCT), nucleic acid sequence-based amplification, rolling circle amplification, recombinase polymerase amplification (RPA), and clustered regularly interspaced short palindromic repeats (CRISPR) [21].

The gold standard among the DNA detection methods to diagnose malaria is widely considered to be the nested-PCR described by Snounou et al. [72,73] targeting the 18S-rRNA gene, including a genus-specific characterized sequence of about 1.2 Kb containing all the *Plasmodium* human-infecting species-specific sequences. A modified nested-PCR assay was developed to improve the original method [74].

Newly developed NAATs include additional target genes, such as mitochondrial DNA (mtDNA), highly sensitive because of the large number of target copies (≈20 copies) [75] and allowing the detection all human *Plasmodium* sp. together with the 18S-rRNA, and other targets, such as *P. falciparum* stevor multigene family [76], telomere-associated repetitive element [77], and *P. vivax* Pvr64 sequence [78].

Several nested-PCR (nPCR) or semi-nested multiplex-PCR (SnM-PCR) are reported in the literature [21]. Small subunit ribosomal RNA (ssRNA) genes are the most used targets of primers used in most of the nested-PCR based assays as such genes are widely used for phylogenetic analysis and are also well characterized from various *Plasmodium* sp. [14]; for the same reasons the highly conserved dpfk13, encoding the Kelch13 protein are also used as target [79]. When morphological problems hinder the identification of malaria parasites at the species level at microscopy nested- and multiplex-PCR results can give an accurate identification [80]. However, these techniques present limitations in their use in low-resource settings or at point-of-need and have some disadvantages: are expensive, time-consuming, require a reliable power supply, require time for sample preparation, reaction set-up (storage of the reagents, separate areas of work to prevent contamination), time to the end of the reaction, and the analysis of the results.

**Figure 4:**
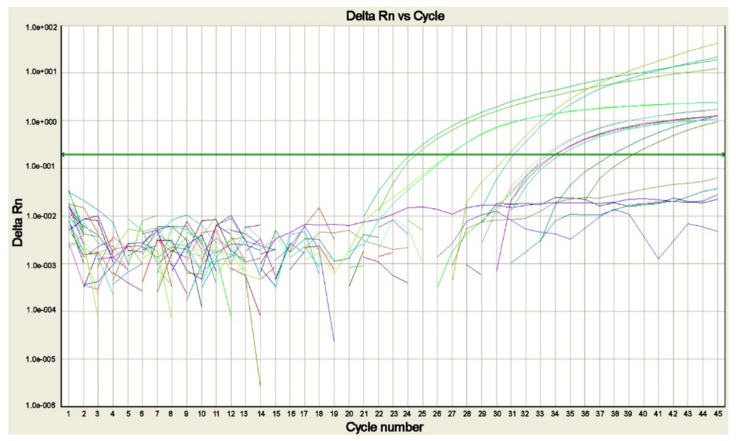
Real-time PCR amplification plot for *Plasmodium* DNA detected in blood samples of patients with suspected malaria. The plot shows the amplification of *P. falciparum*, *P. malariae*, *P. ovale curtisi*, *P. ovale wallikeri*, and *P. vivax* positive controls and of the sample positive for *P. falciparum*, each tested in duplicate (modified from [14]).

Several real-time polymerase chain reaction (real-time PCR) assays to detect and identify the different *Plasmodium* sp. (Figure 4) in a single reaction have been developed to resolve most of the difficulties related to the use of the nested-PCR assays [13,21,27,81,82,83,84].

Real-time PCR is cost-effective, with high sensitivity and specificity, although it is not included among the rapid methods for the initial diagnosis of malaria, it is fast and requires about 1.5 h [14]. Real-time PCR assays are potentially able to detect both low parasitemia levels and mixed infections [85], and for this reason they should be applied not only for the diagnosis but also the prevention of drug-resistant strains from emerging as consequence of misdiagnosis with other methods and related mistreatment and for quality control purposes [86].

The development of commercially available DNA loop-mediated isothermal amplification (LAMP)—based assays is one of the most recent evolutions of DNA amplification assays for the laboratory diagnosis of malaria. It is a simple method based on the isothermal amplification, not requiring special equipment, and producing results that can be read visually or with a real-time turbidimetry. LAMP allows to reduce the time for the result within the recommended 2 h for the diagnosis of malaria and showed to have high efficiency, allowing DNA amplified 10^9^–10^10^ times in 15–60 min [87,88,89]. Advantages of LAMP based assays also include the use of small amounts of blood samples on filter papers and the tolerance of inhibitory substances present in blood samples (hemoglobin/immunoglobulin), but it currently lacks sufficient accuracy [21]. LAMP based assays might be an alternative to the other PCR methods, particularly useful in remote areas because the reaction can simply run in one tube at a constant temperature, not requiring a thermal cycler and producing a rapid malaria diagnosis [90]. Real-time PCR and LAMP assays allow results within a clinically relevant time frame, but they have the same disadvantage common to all NAATs: a positive result can indicate either a current or a recent past infection and cannot be used to differentiate among these two conditions.

Failure in diagnosing malaria with a PCR-based methods can occur when parasites have genetic diversity in the sequences of the target sequence of the primers or when the target gene is present at a very low copy number causing a lower amplification efficiency and consequently a reduced sensitivity [91]. Several quantitative PCR (qPCR) assays have been described to successfully detect *Plasmodium* parasites both in clinical settings and in asymptomatic subjects [92].

It cannot be ignored that molecular assays can detect the parasitic DNA while they are not able to distinguish in the blood sample among some conditions: (i) DNA derived from live parasites, (ii) residual DNA derived from destroyed asexual stage (iii) or circulating gametocytes which can persist after a successful therapy in submicroscopic quantities causing the permanence of DNA up to weeks after a malaria resolved episode. These conditions consequently bring a risk of false positive results producing the recurrence to unnecessary anti-malaria treatment. However, in experimental conditions, the clearance of parasitic DNA from the blood in an animal model was demonstrated within 48 h after malaricide treatment; it can be inferred that detecting *Plasmodium* DNA in a blood sample belonging to a subject with clinical suspicion of malaria could be a sign of active infection, albeit no parasites are revealed at microscopy in the same sample and the result of the NAAT stimulate to repeat blood sampling from the subject to exclude malaria as good practice.

Molecular assays are not indicated for monitoring anti-malaria treatment because in the case of recent or treated infections they can remain positive for up to four weeks (depending on the starting parasitemia), even in absence of viable parasites. Different molecular assays for the diagnosis of malaria, often developed in-house, are widely spread particularly in non-endemic areas stimulating in 2008 the establishment of the WHO International Standard for *Plasmodium falciparum* DNA for NAT-based assays whose use is recommended for the quality control of the reaction and the assessment of the analytical sensitivity of different assays allowing comparative evaluation among their results [93].

Recombinase polymerase amplification (RPA) allows the amplification of single-stranded DNA, double-stranded DNA, methylated DNA, and miRNA [94]. The RPA reaction starts when a recombinase-primers complex is created by the binding of a recombinase protein to primers in presence of ATP and high molecular polyethylene glycol. The combination of the isothermal RPA with the lateral flow detection is an approach to improve molecular diagnostic tools for *P. falciparum* identification in resource-limited conditions. The system requires no or little instrumentation for the reaction as the result can be read-out with the naked eye. The method was demonstrated to be highly sensitive, showing a detection limit of 100 fg and 500 fg, respectively, corresponding to approximately four and 20 parasites/reaction [95]. The RPA reaction allows multiplexing highly depending on target sequences, amplicon size, and primer design [94]. Different detection techniques can also be combined with RPA detection: bridge flocculation assay [21], gel agarose, colorimetric fluorescence [95], quantum dots [96], electrochemical [97] and surface-enhanced Raman scattering detection [98], and for the end-point detection in malaria diagnosis the application of SYBR Green I was also described [99].

The most used in-house and commercially currently available molecular assays are reported in Table 3.

**Table 3:** Different types of molecular assays currently available for the diagnosis of malaria in non-endemic areas.

Molecular Assay	Type of Amplification	Target	Reference
In-house genus/species-specific PCR	Nested-PCR	18S-rRNA	[83,100]
In-house species-specific PCR	Semi nested-PCR	18S-rRNA	[101]
In-house genus/species-specific PCR	QT-NASBA *	18S-rRNA	[102,103]
In-house genus/species-specific PCR	TaqMan	18S-rRNA/mitochondrial DNA sequences	[81,82,83]
In-house genus/species-specific qPCR	TaqMan	18S-rRNA/mitochondrial DNA sequences	[84,104,105,106]
In-house genus/species specific qPCR	Sybr Green	*Pf* CoxI gene *Plasmodium* mitochondrial sequence 18S-rRNA	[107]
Pan and *Pf* Loop AMP^®^ (Eiken Chemical Co., Tokyo, Japan)	Loop mediated isothermal amplification	Mitochondrial DNA sequence	[108,109,110]
RPA	Recombinase polymerase amplification	18S-rRNA	[99]
Molecular-based point of care test	RPA/LAMP	18S-rRNA	[21,111]

* QT-NASBA = Real-Time Quantitative Nucleic Acid Sequence-based Amplification.

#### Molecular-Based Point of Care Test

The use of point of care tests (POCTs) is spread in remote areas having insufficient laboratory infrastructures and not routinely used in malaria non-endemic areas. The POCT should be equipment-free and user-friendly, and they should also be delivered, sensitive and specific, rapid, affordable, and robust [21]. Numerous studies have described the use of nucleic acid testing based-POCT for the detection of *Plasmodium* sp., but a commercial product is not yet available due to technical obstacles such as availability of dedicated thermocycler, optimization of each reaction with suitable materials, and handling of NAATs specific reagents. Isothermal amplification techniques such as LAMP and RPA represent now the most promising techniques to be deeper tested as molecular-based POCTs candidates for the laboratory diagnosis of malaria since they require a simple instrument, using reduced energy and time to achieve a sensitive target detection [111]. Another potential method suitable to be a POCT for the diagnosis of malaria is the microfluidic, either conventional or paper-based assay, which can overcome most of the obstacles in sample preparation, adequate amplification, and detection of genomic targets [21].

### 2.6. Innovative Recently Developed Methods

Recently developed promising methods such as droplet digital PCR (ddPCR) and next generation sequencing (NGS) were proposed to be used in different fields of malaria investigation including basic research and diagnostic purposes. ddPCR is a digital PCR method using a water-oil based emulsion technology providing absolute and direct quantification of a DNA target not requiring a standard curve [112]. ddPCR provides an accurate and absolute quantification by counting the DNA molecules encapsulated in approximately 15,000 discrete, volumetrically defined, water-in-oil droplet partitions that are submitted to endpoint PCR [112]. These techniques were described in laboratory setting in the detection of almost all *Plasmodium* species showing better sensitivity than qPCR.

NGS is a sophisticated method applied to better understand malaria transmission patterns and investigate the malaria parasite movement [113] and for the identification of multidrug resistance related genes in *Plasmodium* species as the major therapeutic barriers that are currently recognized [114].

The miniature direct-on-blood PCR nucleic acid lateral flow immunoassay (mini-dbPCR-NALFIA) is a newly developed and easy-to-use molecular assay proposed for the laboratory diagnosis of malaria in resource-limited settings [115]. Compared to traditional molecular methods, mini-dbPCR-NALFIA is innovative as it does not require DNA extraction and is based on the use of a handheld, portable thermal cycler able to run on a solar-charged power pack or incorporated as a miniature thermal-cycler making the assay well-adapted to resource-limited settings. In addition, for the result read-out a rapid lateral flow strip is used enabling the differentiation of *Plasmodium falciparum* and non-*falciparum* infections.

More recently, an inception-based capsule network was described as an innovative approach to distinguish parasitized and uninfected cells from the analysis of microscopic images [116]. This diagnostic model incorporates neural networks based on inception and imperative capsule networks operating the detection of malaria parasites in microscopic images by classifying them into parasitized and healthy cells. The proposed system is more accurate and faster compared to traditional manual microscopy with an accuracy of 98.10% on the test, while on the 20% split, it achieves an accuracy of 99.3%. These experimental results are encouraging, and the developed model is robust and flexible and has outperformed competing models [116].

“Digital diagnosis” includes the various aspects of digitalization, such as automation in the visualization/analysis of the data deriving from microscopy, RDTs results, and analysis of electronic health records/clinical symptoms using web-based/mobile phone applications, which could enable a graphical user interface and an ease access [117].

Matrix-assisted laser desorption/ionization—time of flight mass spectrometry (MALDI-TOF MS) has brought a revolution in the diagnostic practice for the identification of bacteria and is widely recognized as a method that is fast, and robust, inexpensive, with minimal risk of operator bias. Stauning et al. reported a potential application of MALDI-TOD MS providing proof-of-concept for MALDI-TOF MS-based diagnosis of human malaria [118]. The study concluded that MALDI-TOF MS can be applied to the detection and quantification of *P. falciparum* in human blood albeit not yet applicable to diagnostic practice. Studies on clinical samples together with the development of novel sample processing protocols are required to further develop the method before considering its application to the laboratory diagnosis of malaria.

Recently, Obeng-Aboagye et al. [119] demonstrated that levels of pro-inflammatory cytokines can be used as potential biomarkers for severe malaria, correlating with disease severity. IL-1 βand IL-17A showed good diagnostic potentials and can be considered for use in clinical practice to target treatment.

**Figure 5:**
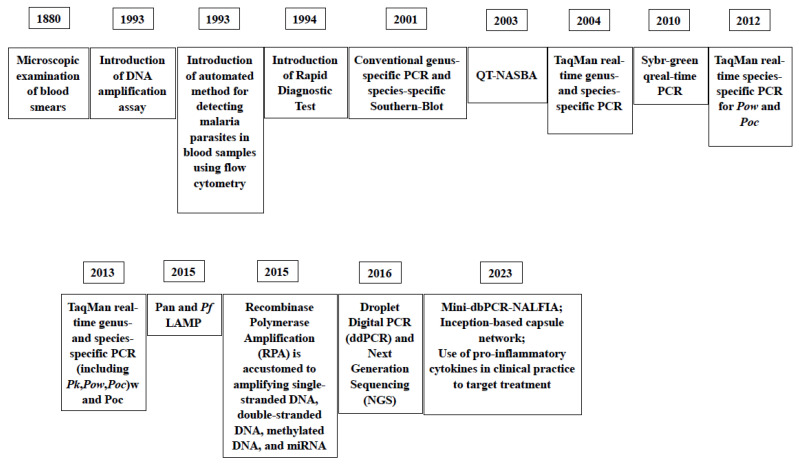
Milestones of the introduction of laboratory assays for the diagnosis of malaria thorough the years [41,72,73,81,82,83,84,108,115,116,119].

In Figure 5, the milestones of the introduction of diagnostic assays for malaria through the years are reported.

## 3. Discussion

In non-endemic areas, malaria cases are mostly classified as imported cases and quite rarely as autochthonous [6]. Malaria is a medical emergency in non-endemic areas, albeit not frequent. A travel history in malaria endemic-areas is the key when malaria is suspected, and malaria diagnosis is mandatory in patients with fever returning from such areas [120]. Malaria clinical presentation lacks specific clinical signs or symptoms, although fever is seen in almost all non-immune patients, and migrants from malaria-endemic areas may have few symptoms [121].

Due to climate change and mass migration people, mostly from endemic areas, indigenous malaria cases are increasingly appearing sporadically (Corsica Italy, Spain) or even as local epidemics (Greece), in countries or regions where the disease was considered eradicated. These aspects should be considered by clinicians in suspecting malaria.

Malaria diagnostics should be performed immediately on suspicion of malaria. Microscopy remains the gold standard for the diagnosis of malaria, due to its high reliability and low cost and being the unique diagnostic assay allowing to indicate an active infection, and that cannot be avoided according to WHO guidelines [70]. However, this method needs stringent prerequisites for both the production and the staining of blood smears of high quality, and the microscopists must be skilled and well trained to achieve the morphological identification and differentiation of the different stages of the different species of malaria parasites, all conditions that are extremely difficult to have and maintain in malaria-free areas [16]. The reliable identification and differentiation of the morphological features of all the developmental stages of infectious *Plasmodium* species can be very challenging, albeit not impossible, even under ideal conditions. Especially as concerns the correct identification of *P. vivax* and *P. ovale*, as these species have very similar morphology of their stages which hinders the differentiation of them. The same obstacle is encountered in distinguishing between *P. knowlesi* and *P. malariae* stages being their differentiation very challenging; *P. ovale wallikeri* and *P. ovale curtisi* are morphologically identical [27,122]. Even for a trained microscopist, it is quite hard to differentiate the atypical morphology of the *Plasmodium* stages as well as to recognize and identify a mixed infection by the microscopic examination of Giemsa-stained blood smears. Moreover, the limit of detection is also low resulting in poor sensitivity because asymptomatic individuals with low sub-microscopic parasitemia may stay undiagnosed and untreated and potentially permit the life cycle of the parasite to spread in the community living in non-endemic areas where the *Anopheles* vectors are present [14].

Since autochthonous malaria cases have been well controlled and imported malaria cases have progressively become rare in non-endemic countries, it is a top-priority to establish an accurate diagnostic method in such settings that has enough sensitivity without the need for trained microscopists. In fact, a fast and accurate diagnostic method can greatly facilitate the early diagnosis of malaria and allows the administration of a timely treatment to an infected patient and effectively reduces mortality related to misdiagnosis that still represents a challenge in non-endemic countries.

The RDTs allow us to obtain rapid results, are simple to use at low cost, and potentially useful in remote areas but lack enough sensitivity and specificity. They have largely expanded the possibility to diagnose malaria, especially in resource-limited regions and in non-immune travelers/tourists to endemic countries. They are a fast and affordable method for malaria diagnosis requiring much less intensive personnel training as compared to microscopy and PCR [28]. However, barriers such as variable sensitivity of the diagnostic assays, regional variation in the genome of the parasite related to gene deletions among the *Plasmodium* species, and a decreased detection of infected subjects, due to the degree of non-*falciparum* malaria related to region where the infection was acquired are reported. HRP2 based RDTs remain the predominant assays that the WHO still recommends due to their quality related to *P. falciparum* detection avoiding misdiagnosis [28]. However, in non-endemic areas, use in combination with RTDs that include PvLDH/PpanLDH or aldolase should be carefully considered when malaria is suspected in a traveler/migrant from Central and South America or from the Indian subcontinent where a high prevalence of *P. vivax* is reported. Anyway, a RDT should not replace microscopy or used alone but might be used in parallel with it [28].

Flow cytometry was successfully proposed for the identification of *Plasmodium* species and quantifying parasitemia, also in cases with low parasite concentrations, but requires well-trained technicians and expensive equipment [47].

Serodiagnosis allows epidemiological surveys, but it is not applicable to the diagnosis of acute malaria [37].

More often in recent years, laboratories have adopted molecular methods for the diagnosis of malaria over traditional methods. As expected, molecular methods have been demonstrated to be at least 10-fold more sensitive than microscopy, proving to be more effective in revealing additional cases of *P. falciparum* including mixed infections missed at microscopy and in differentiating correctly the five species of *Plasmodium* sp. of causing malaria in humans [27,81,82].

In malaria non-endemic areas, PCR-based tests should be the first choice as far as possible, as they are proven to provide additional information (parasite load, species, and resistance) but they require well-trained technicians and a source of energy. PCR (real-time PCR, multiplex PCR, and nested-PCR) brings accurate identification and differentiation of malaria parasites and also have an excellent sensitivity and specificity in detecting low levels of parasitemia [21]. However, such techniques are expensive, time-consuming depending on the method used, require a power supply and are difficult to use in low-resource settings or at the point of need and far from the laboratory.

The isothermal DNA amplification-based methods such as LAMP and RPA are promising methods for diagnostic applications and are the most recent evolutions of DNA amplification methods for malaria diagnosis. Due to these characteristics, LAMP and RPA based assays are simple and fast to use involving low-cost equipment and they might be potentially associated with biosensing technologies for point-of-care diagnostic of malaria also in remote areas. Anyway, their application should be better and more extensively assessed because of false positive results caused by the persistence of DNA of *Plasmodium* species in the blood after a resolved malaria episode might occur [104].

The adoption in diagnostic flow of molecular assays especially in non-endemic settings is encouraged by Dakic and colleagues [104] as complementary methods used together with microscopy, especially in cases of low parasitemia and for *Plasmodium* species identification, considering that most misdiagnosis occur in non-endemic areas in cases of malaria by *Plasmodium* species other than *P. falciparum*. While the molecular assays improved sensitivity and specificity are demonstrated, their selection and inclusion in the malaria diagnostic workflow should be accurately evaluated in each setting. Some laboratories perform the molecular assay when the conventional methods give negative results in subjects with a substantial clinical suspicion of malaria when the *Plasmodium* species cannot be identified.

Molecular assays are generally proposed as confirmatory methods and they are decisive in cases of submicroscopic parasitemia or when mixed infections are suspected and when the morphologic characteristics of the parasite stages overlap, and/or in cases of altered parasite morphology induced by a drug treatment or improper sample handling or storage. It cannot be ignored that they all have the same limit: they are not able to distinguish among DNA derived from live parasites, residual DNA from destroyed parasitic stages or circulating gametocytes which can be still present in submicroscopic trace amounts after successful therapy. As such, the risk of false positive results related to the persistence of trace amount of parasitic DNA after a cured malaria episode, and consequently, unnecessary anti-malaria treatment should be always considered in analyzing the result of such assays [14]. For this reason, the positive result of a molecular assay should be considered together with the clinical condition of the patient and the potential site where the infection may have been contracted allowing this epidemiological analysis to support the results observed.

**Figure 6:**
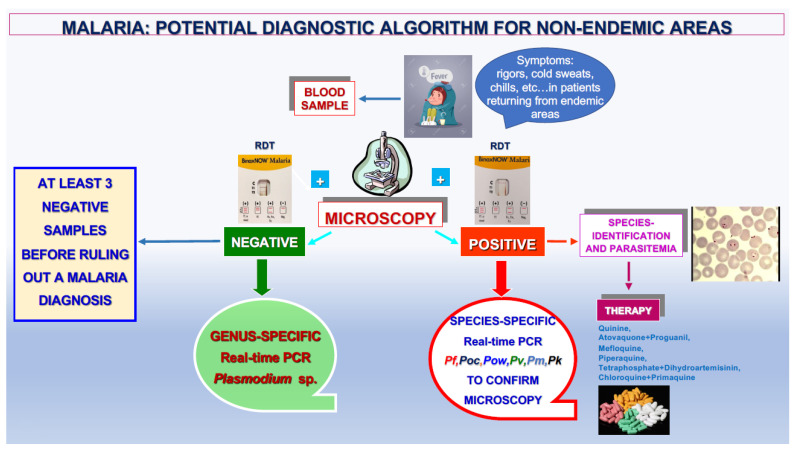
Proposed diagnostic algorithm for malaria in non-endemic areas.

The proposed algorithm (Figure 6) takes into account all these considerations and the decisive role of the genus- and species-specific DNA amplification assays in obtaining an accurate laboratory diagnosis of malaria. According to that reported by the WHO, this diagnostic algorithm can be proposed for both endemic and non-endemic areas based on microscopic examination and the result of RDTs [28].

This review pointed out that diagnostic laboratories located in malaria non-endemic settings can guarantee excellent quality in performing the diagnosis of malaria, with special regard to the identification of *P. falciparum*. In spite of the limitations reported for the methods currently available in the field of malaria diagnosis, they maintain an important role in managing the present global malaria burden, including decreasing its incidence and allowing the adoption of programs for its control.

## 4. Conclusions and Future Directions

Microscopy will remain the method of choice for malaria diagnosis due to its high reliability and low cost even if all innovative methods analyzed in this review offer themselves as a valid support. Diagnostic tools are critical for ensuring the appropriate care of patients with malaria, and in this light, the development of numerous innovations continues and is welcome.

The development of point-of-care testing (POCT) represents the future direction for the diagnosis of the infectious diseases, including malaria, in both endemic and non-endemic settings, according to the WHO global Technical Strategy for Malaria 2016–2030 [9]; it is considered an adequate promising reaction to the need of a prompt diagnosis, together with “on-site” results, which would be an aid for an immediate and accurate anti-malarial treatment and for avoiding the spread of *Plasmodia* among humans and the vector in areas where malaria was eradicated [9]. Moreover, recently published evidence suggested that artificial intelligence can be of aid in assisting pathologists in the detection of malaria parasites and other microorganisms even if at present these tools remain a descriptive step requiring deeper investigation of their application in diagnostic practice [123,124].

Anyway, further studies need to be performed before assessing the automated systems that can be used for routine malaria diagnostic procedures.

## Data Availability

Not applicable.
